# Anticipatory Behavior of the Clonal Plant *Fragaria vesca*

**DOI:** 10.3389/fpls.2018.01847

**Published:** 2018-12-11

**Authors:** Vít Latzel, Zuzana Münzbergová

**Affiliations:** ^1^Department of Population Ecology, Institute of Botany, The Czech Academy of Sciences, Průhonice, Czechia; ^2^Department of Botany, Faculty of Science, Charles University, Prague, Czechia

**Keywords:** anticipatory behavior, foraging, nutrients, light, DNA methylation, epigenetic variation, 5-azacytidine, intelligence

## Abstract

Active foraging for patchy resources is a crucial feature of many clonal plant species. It has been recently shown that plants’ foraging for resources can be facilitated by anticipatory behavior via association of resource position with other environmental cues. We therefore tested whether clones of *Fragaria vesca* are able to associate and memorize positions of soil nutrients with particular light intensity, which will consequently enable them anticipating nutrients in new environment. We trained clones of *F. vesca* for nutrients to occur either in shade or in light. Consequently, we tested their growth response to differing light intensity in the absence of soil nutrients. We also manipulated epigenetic status of a subset of the clones to test the role of DNA methylation in the anticipatory behavior. Clones of *F. vesca* were able to associate presence of nutrients with particular light intensity, which enabled them to anticipate nutrient positions in the new environment based on its light intensity. Clones that had been trained for nutrients to occur in shade increased placement of ramets to shade whereas clones trained for nutrients to occur in light increased biomass of ramets in light. Our study clearly shows that the clonal plant *F. vesca* is able to relate two environmental factors, light and soil nutrients, and use this connection in anticipatory behavior. We conclude that anticipatory behavior can substantially improve the ability of clonal plants to utilize scarce and unevenly distributed resources.

## Introduction

Despite their sessile life style, plants can exhibit very complex and sophisticated behavior for example in foraging for resources, tackling with abiotic or biotic stressors and alike (Karban, 2008). Plant behavior usually reflects actual environmental conditions but mounting evidence demonstrates that behavior of plants can also be strongly affected by their experience with past environments (e.g., Turkington et al., 1991; Galloway and Etterson, 2007; Karban, 2008; Sultan et al., 2009; Whittle et al., 2009; Latzel and Klimešová, 2010; Latzel et al., 2014; Rendina González et al., 2016, 2017). For instance, Gagliano et al. (2014) provided intriguing evidence that *Mimosa pudica* trees can store memory on a false disturbance stimulus, which enables them to ignore the stimulus for relatively long period but being still able to respond to other relevant stimuli. It has also been shown that plant behavior can be in some cases explained better by their past environmental interactions than by their actual environment (Münzbergová and Hadincová, 2017). The memory of the past environments cannot only modify actual plant behavior, but it can also enable plants to anticipate future conditions (Karban, 2008; Calvo and Friston, 2017). For example, plants are able to adjust photosynthetic apparatus in anticipation of future light quality changes by use of memory on former light quality (Karpinski and Szechynska-Hebda, 2010; Shemesh et al., 2010). Plants can also modify their growth according to expected future competition for resources based on their previous experience with the light quality (Gagliano et al., 2016; Novoplansky, 2016). These are but a few examples describing the ability of plants to memorize past experiences and apply the memory in actual and anticipatory behavior.

Recent study also provided striking evidence that plants can use their memories for associative learning, an ability that has been previously ascribed only to animals (Watanabe et al., 2001; Gil et al., 2007; Shettleworth, 2007; Nilsson et al., 2008; Molet and Miller, 2014) or sophisticated man-made machines or software (e.g., Goldschmidt et al., 2014). Associative learning, the ability to place different stimuli into a functional context, is thought to be enabled by complex neural system (Trewavas, 2003, 2017), which is obviously not present in plants. Despite this, Gagliano et al. (2016) showed that pea plants can associate location of light source with an air flow, i.e., with a neutral cue. After a learning period and when light source was removed, plants grew toward or opposite to the air flow due to anticipation of light source in different air flow regime (Gagliano et al., 2016). They therefore suggest that associative learning is an essential component of plant behavior.

Latzel et al. (2016) proposed that anticipatory behavior can be ecologically important particularly for clonal plants. Many clonal plants are able to move over large area by producing ramets connected by stolons or rhizomes. Such movement provides clonal plants with the ability to reach distant resources as well as escape from unfavorable conditions. Since resources occur mostly in patches and random clonal growth would not be efficient in their effective utilization, clonal growth can be targeted allowing to actively forage for unevenly distributed resources (Grime and Mackey, 2002; de Kroon and Mommer, 2006). Foraging is enabled by the plants’ ability to perceive quality of patches and make decisions between patches of different qualities (e.g., Bell, 1984; Waters and Watson, 2015). In the case the information on spatial and temporal variation of the environment is stored and shared among interconnected ramets of a clonal plant, it can be translated into better understanding of the environmental heterogeneity resulting thus in the ability of a clone to predict future resource patches (Latzel et al., 2016). Memories on the environmental interactions can be stored in the form of epigenetic change. Epigenetic change should be well maintained in clonal plants because of the lack of meiosis during clonal reproduction as meiosis represents a barrier for most environmentally induced epigenetic changes (reviewed by Paszkowski and Grossniklaus, 2011).

**FIGURE 1:**
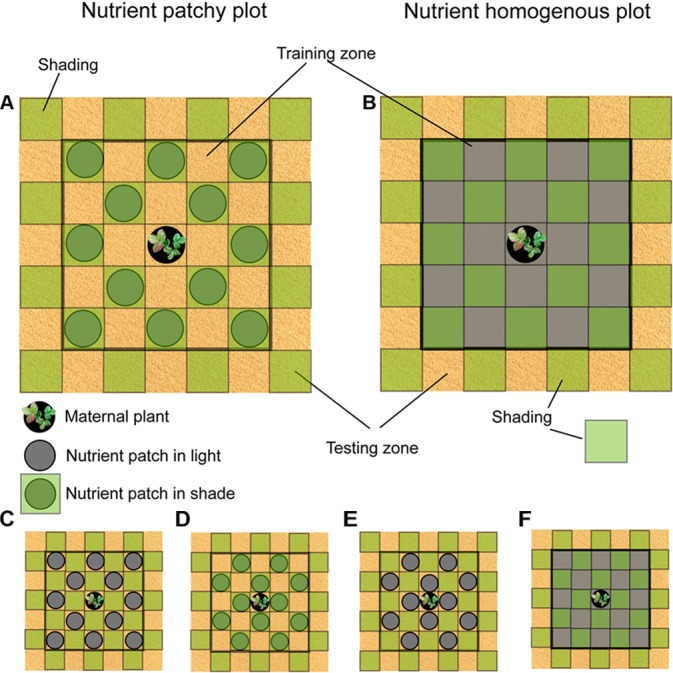
Scheme of the study design. Circles represent nutrient patches. Green squares represent shading by the textile. **(A)** Nutrient patches in shade, light patches in a cross pattern. **(B)** Nutrient homogenously distributed, light patches in a cross pattern. **(C)** Nutrient patches in full light, light patches in a diagonal pattern. **(D)** Nutrient patches in shade, light patches in a diagonal pattern. **(E)** Nutrient patches in full light, light patches in a cross pattern. **(F)** Nutrient homogenously distributed, light patches in a diagonal pattern.

For the purpose of this study, we selected *Fragaria vesca* as a model. *F. vesca* is a clonal plant with extensive stolons adapted to a wide range of patchy environments. The species can actively forage for soil nutrients by directional placement of ramets into high quality patches (Waters and Watson, 2015). We hypothesized that clones of *F. vesca* are able to memorize position of soil nutrients with particular type of light intensity (full light or shade). Such memory will consequently enable clones to predict future nutrient positions based solely on light intensity of the newly occupied environment. In order to test the hypothesis, we grew *F. vesca* in nutrient homogenous and nutrient patchy environments. At the same time, all plants also experienced patches of full light and shade. In the case of the nutrient patchy environment, nutrient patches were located either in full light or in shade (further referred to as a *training zone* in the text, see also Figure 1). After a period of training, all clones reached a *testing zone* (see Figure 1) that consisted of full light or shade patches only, i.e., without soil nutrients. We predicted that *F. vesca* in the testing zone will grow preferentially into the light intensity corresponding to the nutrient location in the training zone. Plants that experienced nutrient homogenous environment in the training zone will grow preferentially into the light patches in the testing zone. Moreover, in order to test the role of epigenetic memory in the potential anticipatory behavior, we altered methylation status of a subset of the plants by their repeated spraying with 5-azacytidine solution (Puy et al., 2018). Such demethylation effectively erases memories on past environmental interactions in clonal plants (Rendina González et al., 2016; Puy et al., 2018). We expected that experimentally demethylated plants will not exhibit anticipatory behavior or the level of anticipatory behavior will be altered. In addition, because plants’ ability to utilize soil nutrients is affected by the light availability (Hutchings and de Kroon, 1994), we also hypothesized that foraging for soil nutrients in the training zone will be affected by the light intensity of the nutrient patches.

## Materials and Methods

### Species

*Fragaria vesca* L., Rosaceae, is a perennial clonal plant that grows in disturbed and degraded forests, forest edges, and meadows. Seeds of *F. vesca* were provided by local seed producer, Planta Naturalis^1^. Plants were pre-cultivated in a greenhouse from February to March 2017 (12 h/12 h day/night regime with temperature regime of 22°C/15°C) before they were moved to a garden experiment.

### Garden Experiment

The study was performed in a garden of the Institute of Botany of the Czech Academy of Sciences in Průhonice, Czechia (N 49°99′46″, E 14°56′62″) from April to August 2017. The plants were grown in 85 **plots** of 105 cm × 105 cm (distance between plots was around 50 cm) that were distributed in 8 beds (18 m × 1.5 m × 10 cm, the bottom of the beds was isolated with geotextile to prevent roots from reaching nutrients under the beds) filled with sand. All plots were divided into 49 square subplots (15 cm × 15 cm), further referred to as **patches** (see Figure 1). Nutrients were located in the central part of each plot (**training zone** consisting of 24 patches, 5 × 5 patches except the central one, Figure 1). The 24 patches located at the edge of the plot were kept without nutrients (**testing zone,** Figure 1). Plots were assigned to two different soil nutrient distributions: **patchy** and **homogenous**. Nutrients were placed in 12 out of the 24 patches in the training zone in each patchy plot in two possible regular patterns, further referred to as diagonal (Figure 1A) and cross (Figure 1D) nutrient pattern. Both patterns were used in order to account for different distance of the patches from the maternal plant located in the center of each plot. Nutrient patches were created by plastic pots (15 cm in diameter, 5 cm high) filled with standard potting substrate (NH_4_: 200 mg/l; NO_3_: 100 mg/l; P: 60 mg/l; K: 5000 mg/l; Mg: 250 mg/l; Ca: 200 mg/l) buried into each patch. **Homogenous plots** were created by replacing sand from the 24 patches in the training zone (Figure 1B) with a mixture of sand and standard potting substrate of the volume of 12 pots, i.e., the same volume of potting substrate as used in the patchy plots. All plots were covered with a green textile (woven fabrics) from 7 cm height. The textile that reduced light by 65% but did not alter light spectrum had 15 × 15 cm openings in the same pattern distribution as the soil nutrient patches, i.e., cross or diagonal pattern. In the case of nutrient patchy plots, light patches either matched nutrient patches (Figures 1C,E) or not (Figures 1A,D). Cross (*N* = 14) and diagonal (*N* = 14) light patches patterns were also distributed in the nutrient homogenous plots (Figures 1B,F). Light patches were distributed also in the testing zone without soil nutrients (Figure 1). Single pot filled with standard potting soil and planted seedling of *F. vesca* (maternal plant) was placed in the central light patch of all plots (Figure 1). The design of the study resulted in 57 plots with patchy nutrient distribution where 29 plots had nutrients located in shade (15 in cross pattern and 14 in diagonal pattern) and 28 plots had nutrients located in light (14 in cross and 14 in diagonal pattern). Twenty-eight plots had nutrients distributed homogenously.

### Experimental Demethylation

Plants in five plots per nutrient distribution and light pattern (30 plants in total) were sprayed two times a week with 100 μmol aqueous solution of 5-azacytidine and surfactant from May 1st to June 15th 2017. Such an approach has the same demethylation capacity as the traditional demethylating method based on germination of seeds in 5-azacytidine solution but has no unwanted side effects that are commonly observed in the traditional method (Puy et al., 2018). It has also been shown that regular spraying of clonal plants with 5-azacytidine can remove epigenetically driven transgenerational effects (Rendina González et al., 2016), namely epigenetic memory on past environmental interactions. The remaining 55 plants were sprayed with water and surfactant to account for potential effects of the surfactant on the plant behavior.

### Measurements

Plants were harvested from July to August 2017 when at least three patches of the testing zone were occupied. Before the harvest, we recorded the number of rooting and non-rooting ramets in each patch in the testing zone and number of all ramets (rooting and non-rooting) in all the other patches. Consequently, above ground biomass in each patch was harvested, dried at 60°C for 48 h and weighed. We consider the number of ramets as the measure of foraging behavior in *F. vesca* and therefore are using the number of ramets as the primary value in our study. Moreover, we consider rooting ramets as the best representative of anticipatory behavior in our study as it is clear that the rooted ramets will remain in observed patches whereas non-rooting ramets can still be moved to different patches via movement of stolons. We also provide results for above ground biomass as this is often considered as a proxy of plants fitness in clonal plants.

### Statistical Analyses

The statistical analyses were done separately for the training zone and the testing zone. In both, the training and the testing zone, we first analyzed only the data with patchy nutrient distribution in the training zone. For the testing zone, we then also separately compared the nutrients in the light treatment and nutrients in the shade treatment to the homogenous treatment.

The data were analyzed using mixed-effect models with heterogeneity type (homogenous, nutrients in light, nutrients in shade), 5-azacytidine application (yes/no) and light in individual patch (yes/no) and all their interactions as predictors and plot code as a random factor. Day of harvest and patch distance from the central ramet were used as covariates in all cases. For the tests in the testing zone, we also used number of ramets or biomass in the training zone as additional covariate to account for size differences between the plants entering the testing zone.

The dependent variables were number of all ramets and number of rooting ramets (following Poisson distribution) and total biomass in a patch (+0.01, following Gamma distribution). Significance of each term was assessed using Akaike information criteria by comparing a full model with a model excluding the tested term resulting in a test with type II sum of squares (Crawley, 2012). Reduction in the AIC value in the full model compared to the reduced model by at least -1.8 was used as a measure of significance of the given variable. We also derived the corresponding *p*-values.

For pair-wise comparison of the different treatments shown in graphs, we calculated marginal effects and their associated *p*-values using the package Margin implemented in R. The values express the proportional change in the response variable (e.g., number of ramets and biomass) between the two categories being compared, after accounting for all the other variables entering the corresponding models (plot position, distance from maternal ramet, and day of harvest). All the analyses were performed using lme4 package (Bates et al., 2015) in R 3.4.2 for Windows (R Development Core Team, 2011).

## Results

### Foraging for Resources: Patchy Plots and Training Zone Only

**FIGURE 2:**
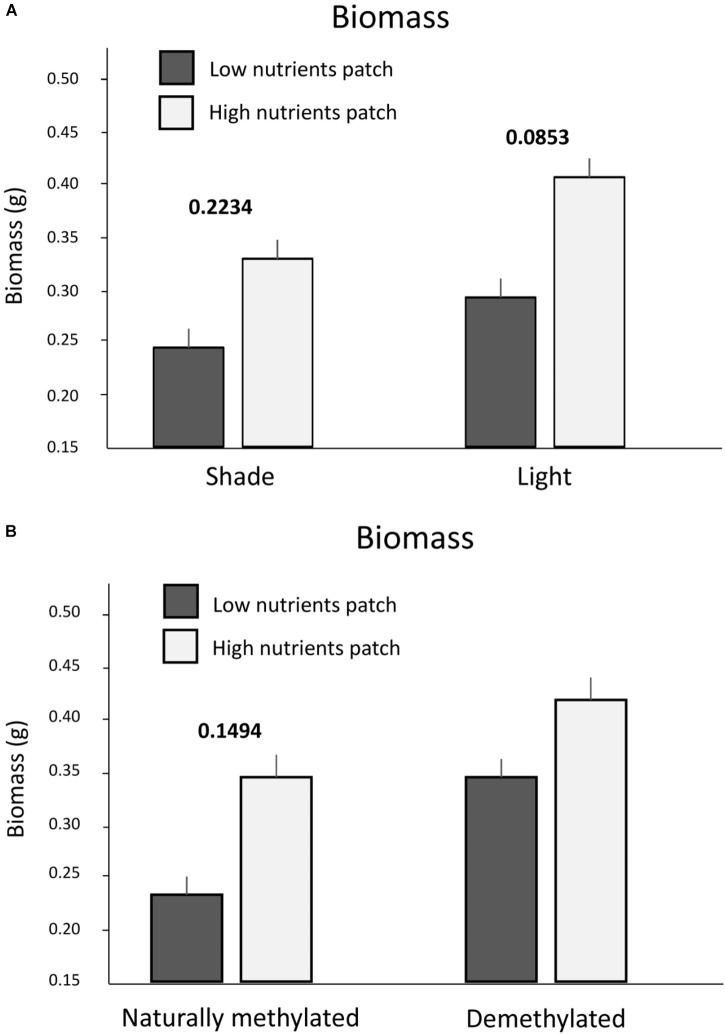
Biomass production in the training zone of nutrient heterogeneous plots. Presented are only significant interactions between nutrients and light intensity. **(A)** Biomass production in shade and light and nutrient rich or poor patches. **(B)** Biomass production in nutrient rich or poor patches of naturally methylated and experimentally demethylated clones. Means and SE are shown. Above each pair of columns, we show the values of significant (*p* < 0.05) in bold or marginally significant (*p* < 0.1) in italics pair wise marginal effects estimated after accounting for all the other variables entering the corresponding models, see also Section “Materials and Methods.” No value above a pair of columns means no significant marginal effect.

Number of ramets and biomass were generally higher in nutrient rich patches and in full light. However, the effect of nutrients on the biomass production interacted with light intensity (Table 1 and Figure 2A). The difference between nutrient rich and poor patches was higher if the nutrients were located in full light (Figure 2A). Experimental demethylation reduced the differences in biomass production between patches of contrasting nutrients (with and without nutrients, Figure 2B).

**Table 1:** Training zone only: nutrient patches in the light or shade.

		Number of all ramets		Biomass	
			
	*Df* error	ΔAIC	*P*	ΔAIC	*P*
Harvest day	52	**-27.4**	**<0.001**	**-76.93**	**<0.001**
Distance from mother	1303	**-6.6**	**0.003**	**-96.01**	**<0.001**
Nutrient patch (N)	52	**-8.3**	**0.001**	**-29.5**	**<0.001**
Light intensity (L)	1303	**-112.4**	**<0.001**	**-4.68**	**0.009**
Azacytidine (A)	52	-0.7	0.102	1.22	0.377
N × L	1303	1.9	0.719	**-2.09**	**0.043**
L × A	1303	1.4	0.407	0.95	0.305
N × A	52	-1.6	0.054	**-5.45**	**0.006**
N × L × A	1303	-0.4	0.124	-0.06	0.151


*Results of linear mixed-effect models testing the effects of nutrient patch (i.e., nutrients in light or in shade), light intensity of a patch (light or shade), 5-azacytidine application (yes/no) and their interactions on biomass and number of ramets produced in each patch in the training zone. Plot code was used as a random factor in the models. Harvest date and patch distance from the mother, i.e., from the central patch, were used as covariates. The results show the ΔAIC values indicating the loss of information after excluding the given term from the model, and the corresponding *p*-values. Statistically significant results are in bold face. Df error are estimated based on the level of replication for the given variable.*

### Anticipatory Behavior: Patchy Plots and Testing Zone Only

**FIGURE 3:**
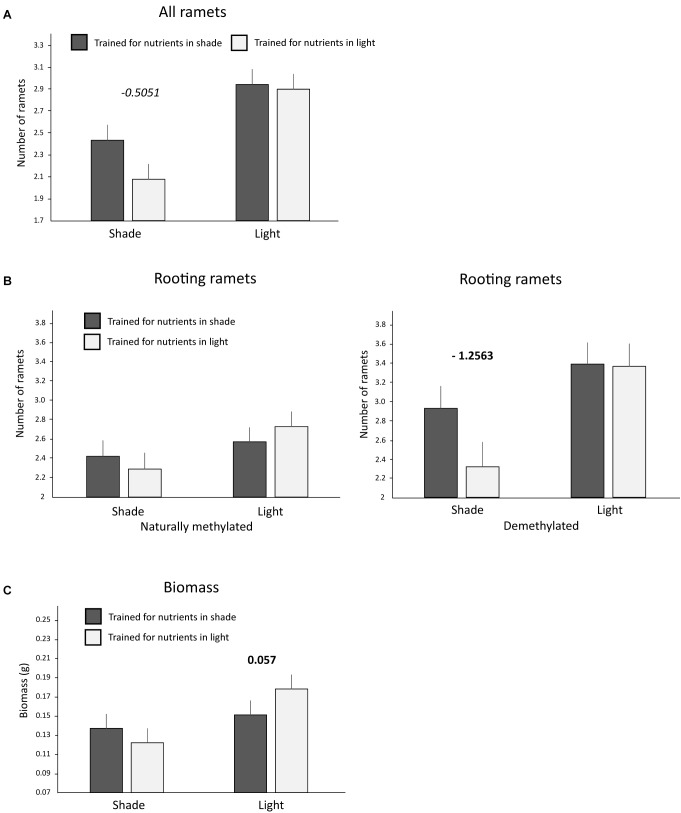
Ramet and biomass production in the testing zone of nutrient heterogeneous plots. Presented are only significant interactions between training history and actual light intensity. **(A)** Number of all ramets of clones trained for nutrients to occur in shade or light. **(B)** Number of rooting ramets of clones trained for nutrients to occur in shade or light of naturally methylated and demethylated clones. **(C)** Biomass production of clones trained for nutrients to occur in shade or light. Significant interaction between training history (trained for shade/light) and actual light conditions (shade/light) represents significant difference in response to actual light intensity depending on previous training history, i.e., evidence of anticipatory behavior. Means and SE are shown. Above each pair of columns, we show the values of significant (*p* < 0.05) in bold or marginally significant (*p* < 0.1) in italics pair wise marginal effects estimated after accounting for all the other variables entering the corresponding models, see also Section “Materials and Methods.” No value above a pair of columns means no significant marginal effect.

Significantly more ramets and biomass was produced in the testing zone by clones that had been trained for nutrients in the light compared to clones trained for nutrients in the shade (Table 2). Number of ramets (all and rooting only ramets) as well as biomass in the testing zone were significantly higher in light than in shade. The anticipatory behavior can be detected as a significant interaction between training (Trained Nutrients) and actual light conditions (light/shade). Such interaction reveals whether plants placed their ramets to shade or light differently, based on their previous training, e.g., more to shade than to light in case that were trained for nutrients to occur in shade. Indeed, the number of ramets as well as biomass were significantly dependent on the training history of the clone (interaction Trained Nutrients × Light Intensity, Figure 3 and Table 2). Clones that had been trained for nutrients in shade placed more ramets (all ramets, marginally significant pair wise marginal effect) into shade compared to clones trained for nutrients in light, while there was no such difference in light patches (Table 2 and Figure 3A). Clone tended to produced more biomass in shade than in light if it was trained for nutrients in shade (nonetheless, the pair-wise marginal effect is not significant, Figure 3C). Similarly, clone produced more biomass in light than in shade if it was trained for nutrients in light (significant pair-wise marginal effect, Figure 3C). Experimental demethylation had a significant interactive effect on anticipatory behavior in the number of rooting ramets (interaction 5-Azacytidine × Trained Nutrients × Light Intensity, Figure 3B and Table 2). Interestingly, experimental demethylation increased the number of rooting ramets in shade patches in clones that had been trained for nutrients to occur in shade compared to naturally methylated clones (significant pair-wise marginal effect, Figure 3B). On the other hand, experimental demethylation reduced the difference in the number of rooting ramets in light patches between clones trained for nutrient positions in shade or light compared to naturally methylated clones (Table 2 and Figure 3B).

**Table 2:** Testing zone only: the effect of previous training for nutrients to occur in shade or light on the behavior in light and shade patches without presence of nutrient patches.

		Number of all ramets		Number of rooting ramets		Biomass	
				
	*Df* error	ΔAIC	*P*	ΔAIC	*P*	ΔAIC	*P*
Harvest day	52	1.2	0.373	**-7.2**	**0.002**	**-36.9**	**<0.001**
Clone biomass	52	**-21.5**	**<0.001**	**-11**	**<0.001**	**-11.7**	**<0.001**
Distance from mother	1303	**-67.1**	**<0.001**	**-106.9**	**<0.001**	**-29.6**	**<0.001**
Trained nutrients (N)	52	0.4	0.21	0.5	0.225	**-1.7**	**0.050**
Light intensity (L)	1303	**-55.5**	**<0.001**	**-73.9**	**<0.001**	**-9.7**	**0.001**
Azacytidine (A)	52	1.1	0.355	1.6	0.517	1.6	0.518
N × A	52	1.3	0.393	1.3	0.388	-0.2	0.135
L × A	1303	1.3	0.384	1.1	0.327	1.6	0.52
N × L	1303	**-2.9**	**0.027**	-0.1	0.146	**-2.3**	**0.037**
N × L × A	1303	0.5	0.221	**-1.8**	**0.053**	-0.8	0.097


*Results of linear mixed-effect models testing the effects of trained nutrients (i.e., nutrients in light or in shade in the training zone), light intensity of a patch (light or shade), 5-azacytidine application (yes/no) and their interactions on biomass and number of ramets produced in each patch in the testing zone. Plot code was used as a random factor in the models. Harvest date, distance from the mother, i.e., from the central patch, and clone biomass in the training zone were used as covariates. The results show the ΔAIC values indicating the loss of information after excluding the given term from the model, and the corresponding *p*-values. Statistically significant results are in bold face. *Df* error are estimated based on the level of replication for the given variable.*

### Comparison of Patchy and Homogenous Plots: Testing Zone Only

**FIGURE 4:**
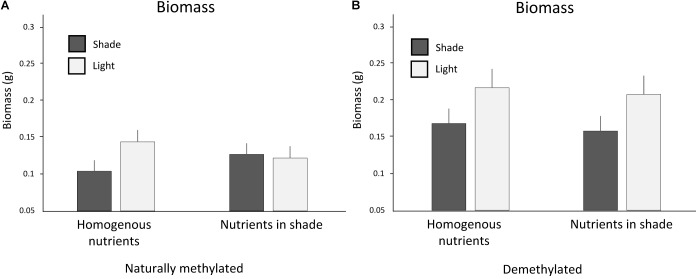
Biomass production in the testing zone of clones **(A)** trained for nutrients in light or homogenously distributed nutrients and **(B)** naturally methylated and demethylated clones trained for nutrients to occur in shade or homogenously distributed nutrients. Means and SE are shown.

Clones that experienced homogenous nutrients produced slightly more biomass than clones trained for nutrients in shade (Table 3). Response to light between clones trained for nutrients to occur in shade or light and clones with homogenous soil nutrients interacted with application of 5-azacytidine (interaction Trained Nutrients × Light Intensity × 5-Azacytidine, Table 3 and Figure 4). Naturally methylated clones trained for nutrients in shade patches produced slightly more biomass in shade than in light in the testing zone whereas naturally methylated clones that experienced homogenous distribution of soil nutrients produced more biomass in light than in shade (Figure 4). On the other hand, demethylated clones produced always more biomass in light than in shade in the testing zone independent of the distribution of the nutrients in the training zone.

**Table 3:** Testing zone only: comparisons of training for nutrients to occur in shade or light with homogeneous nutrient distribution (i.e., trained for nutrients occurring both in shade and light patches).

		Homogenous vs. trained nutrients in shade						Homogenous vs. trained nutrients in light					
			
		Number of all ramets		Number of rooting ramets		Biomass		Number of all ramets		Number of rooting ramets		Biomass	
							
	Df error	ΔAIC	*P*	ΔAIC	*P*	ΔAIC	*P*	ΔAIC	*P*	ΔAIC	*P*	ΔAIC	*P*
Harvest day	51	**-14.2**	**<0.001**	**-35.3**	**<0.001**	**-41.6**	**<0.001**	**-5.7**	**0.005**	**-20.8**	**<0.001**	**-34.9**	**<0.001**
Clone biomass	51	**-54.8**	**<0.001**	**-27**	**<0.001**	**-2.9**	**0.023**	**-42.4**	**<0.001**	**-27**	**<0.001**	**-1.3**	0.068
Distance from mother	1302	**-45.6**	**<0.001**	**-84.9**	**<0.001**	**-17.9**	**<0.001**	**-92.3**	**<0.001**	**-136.9**	**<0.001**	**-24.3**	**<0.001**
Trained nutrients (N)	51	0.3	0.19	1.3	0.402	**-3.8**	**0.015**	1.9	0.711	1.8	0.61	1.2	0.362
Light intensity (L)	1302	**-41.7**	**<0.001**	**-53**	**<0.001**	**-3.8**	**0.016**	**-72.5**	**<0.001**	**-72.6**	**<0.001**	**-18**	**<0.001**
Azacytidine (A)	51	**-4.8**	**0.009**	-1.1	0.081	2	0.798	**-1.8**	**0.05**	-0.5	0.112	0.1	0.172
N × A	51	1.9	0.757	2	0.971	0.7	0.261	2	0.908	2	0.908	1.7	0.634
L × A	1302	1.9	0.77	1.7	0.618	1.9	0.834	1.2	0.365	-0.1	0.151	0.2	0.186
N × L	1302	0.3	0.196	1.9	0.871	1.4	0.461	1.3	0.401	0.4	0.202	-0.5	0.119
N × L × A	1302	2	0.997	1.9	0.717	**-2**	**0.046**	0.6	0.233	-0.2	0.135	2	0.818


*Results of linear mixed-effect models testing the effects of trained nutrients (i.e., nutrients in light or in shade in the training zone) compared to homogenous nutrient distribution, light intensity of a patch (light or shade), 5-azacytidine application (yes/no) and their interactions on biomass and number of ramets produced in each patch in the testing zone. Plot code was used as a random factor in the models. Harvest date, distance from the mother, i.e., from the central patch, and clone biomass in the training zone were used as covariates. The results show the ΔAIC values indicating the loss of information after excluding the given term from the model, and the corresponding *p*-values. Statistically significant results are in bold face. Df error are estimated based on the level of replication for the given variable.*

## Discussion

Active foraging for unevenly distributed resources (Bell, 1984; Waters and Watson, 2015), division of labor (Alpert and Stuefer, 1997) and resources and/or information exchange between ramets (Fischer and Stöcklin, 1997; Gómez et al., 2007; Louapré et al., 2012) are among the most commonly mentioned ecologically and evolutionarily beneficial features of clonal plants. It has also recently been shown that epigenetic memory of past environments is likely another mechanism that provides clonal plants with an advantage in tackling ambient environment (Rendina González et al., 2016, 2017). Here we provide new evidence that clones of *F. vesca* are able to anticipate nutrient conditions of not yet occupied environment thanks to the ability to associate position of nutrient patches with particular light intensity. We suggest that such anticipatory behavior increases their ability to utilize unevenly distributed and limited resources.

Our results show that clones of *F. vesca* have developed an association that nutrient patches are located in environments of particular light intensity. When clones reached the testing zone without nutrients, they could place ramets either to shade or light. Considering the fact that growth toward light is an essential feature of all plants and that there were no volatiles indicating particular position of soil nutrients, the intuitive expectation would be that clones preferentially place their ramets into the light patches. Indeed, we observed that light patches were occupied more often than shade patches in the testing zone irrespectively of their training history. However, at the same time there was also visible and significant shift (demonstrated as significant interaction in training history vs. actual light intensity) in placing ramets into shade or light based on the previous experience of the clones. The clones placed ramets to shade more often if trained for nutrients to occur in shade if compared with clones trained for nutrients in light (see Figures 3A,B). The clones also tended to place rooting ramets more in the light patches if trained for nutrients to occur in the light and produced more biomass in light patches in comparison to plants trained for nutrients to occur in shade (see Figure 3C). Light is an essential resource for plants and plants therefore placed large fraction of their ramets to light even in case of anticipation of nutrients in shade. Nonetheless, even small increase in placement of ramets into shade than into light, as observed in our study, can significantly improve soil nutrients uptake. These nutrients can be distributed over the clone and costs of reduced photosynthesis due to the shade can be outweighed by benefits of acquired soil nutrients. We therefore consider such behavior supported by significant interaction of trained nutrients and light as a clear evidence that experience of the clones in the training zone established an association of nutrients with particular light intensity.

Our study is not the first to demonstrate association between two environmental factors in an organism without neural system. Gagliano et al. (2016) demonstrated that pea plants can associate light source with an air flow direction. They chose foraging for light because light is absolutely essential for plants and provides them significant evolutionary advantage (Gagliano et al., 2016). We applied more complicated design as some clones were challenged to forage either for light or soil nutrients if these were located in shade. Clonal plants can share vital resources between ramets supporting thus those that experience low resources availability (Stuefer and Hutchings, 1994; Alpert, 1996; de Kroon et al., 1996). Hence, contrary to non-clonal plants, clonal plants can theoretically distribute ramets among patches of different resource types in order to better utilize all available resources. Such behavior could be considered as a form of division of labor (Alpert and Stuefer, 1997). Therefore, when a clonal plant is facing the dilemma whether to forage for soil nutrients or light, it can effectively do both. *F. vesca* occupies mostly biotopes with irregular disturbance events. In such biotopes nutrient patches can occur both in *open environment* due to recent disturbance that removed above ground biomass and enriched soil via decaying organs as well as *in shady environment* because of the absence of disturbance and vigorous vegetation growth due to high soil nutrient availability. Assuming that the light intensity and quality of available patches can be sensed from much longer distance (altered R: FR ratio of reflected light from surrounding vegetation, Franklin, 2008) than their soil nutrient status, the anticipatory behavior can enable a clone to avoid or reduce shade avoidance, a common attribute of plants (de Kroon and Hutchings, 1995), and place some of its ramets into shaded but nutrient rich patches. These ramets can thus specialize more on soil nutrients uptake and less on photosynthesis.

By comparison of the behavior between clones with homogenously distributed soil nutrients and clones trained for nutrients to occur in shade or light environment we intended to test whether association between soil nutrients and light intensity was established either in shade or light or in both types of light intensity patches. We considered growth of clones experiencing nutrient homogenous environment as a standardized behavior where no association was possible. However, statistical tests revealed mostly non-significant differences between behavior of clones experiencing nutrients in shade or light from the clones experiencing homogenous nutrient distribution. The marginally significant difference in biomass between clones trained for nutrient in light and clones that experienced homogenous nutrients could suggest that association occurred particularly if nutrients were located in light. On the other hand, the interactive response to 5-azacytidine (Figure 4) showed that naturally methylated clones produced slightly more biomass in shade than in light if they were trained for nutrients to occur in shade in comparison to clones that experienced homogenous nutrients and the effect disappeared after demethylation. Such results suggest that association between nutrients and different light intensity had been established both in shade and in light. The stronger association after training for nutrients in shade than in light, compared to homogenous training zone, is in line with the fact that shade is naturally avoided by most species. As a result, clones trained for nutrients in light do not really alter their behavior compared to controls, while clones trained for shade do.

### Potential Mechanisms Enabling Anticipatory Behavior in *F. vesca*

de Kroon et al. (2009) proposed that a growing ramet integrates local information on environmental quality of the ramet with the information on environmental qualities of all the other ramets of the clone. In other words, response of a ramet to the environment is dependent also on the environment experienced by the other interconnected ramets. Such multisensory integration can thus help clonal plants in optimizing their architecture in situations when a single cue does not provide enough information about the full environmental context (Louapré et al., 2012; Latzel et al., 2016; Casal and Questa, 2018). Plant response to light is regulated by interplay of three major phytochromes (phyA, phyB, and phyC) and phytochrome interacting factors (PIFs) (Duek and Fankhauser, 2005; Monte et al., 2007). It has been shown that prolonged expression of PHYA gene that encodes phyA suppresses shade-avoidance responses in transgenic tobacco plants (McCormac et al., 1991, 1992). Shade-induced growth is also enabled by expression of genes activated by PIFs (Franklin, 2008; Lorrain et al., 2008; Li et al., 2012; Yang and Li, 2017). It is thus possible that ramets that encountered nutrients in shade have intensified activity of phyA and PIFs derived proteins and hormones and these chemicals were spread over the clone. This could promote shade-induced growth of other interconnected ramets. Alternatively, epigenetic modification of DNA may be involved in associative learning of plants (Gagliano et al., 2016; Latzel et al., 2016). In our study, epigenetically modified expression of PIFs and PHYA could be passed to offspring ramets during clonal growth, which could lead to enhanced shade-induced growth of all newly emerging ramets. Some evidence that *F. vesca* response to light and/or shade is to some degree epigenetically regulated was provided by the growth of experimentally demethylated clones. We expected that demethylation will reduce the effect of anticipatory behavior because of the alteration of memories on past environment (e.g., Rendina González et al., 2016; Puy et al., 2018). Nonetheless, we observed exactly the opposite effect of demethylation in clones trained for nutrient position in shade. In these clones, demethylation enhanced their shade-induced growth (Figure 3B). This response suggests that demethylation reduced shade-avoidance and promoted placement of ramets into shade, however, only if plants were trained for nutrients in shade. This suggests that DNA methylation can play some role in plastic response of clones to different light intensity with consequent effect on association of nutrient occurrence with shade. Similar mechanisms could potentially enable association of full light conditions with nutrient position. In this case, clones can be hormonally and/or epigenetically adjusted to increased shade-avoidance, which can result in increased placement of ramets to full light patches.

The inevitable prerequisite for the proposed mechanisms potentially enabling anticipatory behavior is that regulatory mechanisms of foraging for nutrients and light are not fully independent of each other. It is known that the combination of information on light quality and temperature enables plants to optimize physiology and phenotype (Casal and Questa, 2018). We suggest that similar interplay between information on the light intensity and nutrient quality that alter plant phenotype can also occur in clonal plants.

## Conclusion

Anticipatory behavior can improve utilization of limited and unevenly distributed resources by clonal plants due to better understanding of environmental variability and heterogeneity. We propose that the observed association of nutrient position with particular light intensity that enables anticipatory behavior can be established thanks to coordination of molecular mechanisms regulating foraging for light and nutrients. Therefore, anticipatory behavior can be established on the basis of a joint regulation of existing molecular regulatory mechanisms and does not require any other mechanisms such as neural system known in animal kingdom. In line with Gagliano et al. (2016) our results suggest that anticipatory behavior is an important component of plant behavior. We also suggest that epigenetic memory could be one of the mechanisms enabling anticipatory behavior. Nonetheless, we are aware that we would need to employ NGS molecular methods and analyze DNA regions associated with genes involved in response to light and nutrients to get better idea about the role of epigenetic regulation in anticipatory behavior.

Our study also supports the evidence that *F. vesca* is able to actively forage for soil nutrients as was demonstrated by Waters and Watson (2015) and that the foraging ability is likely also epigenetically coordinated. We also provided evidence that foraging for nutrients is not independent from the light intensity of nutrient patches. This interaction should be considered in future ecological research. Future research should also evaluate how common the anticipatory behavior is among other clonal species with different growth strategies, e.g., guerrilla vs. phalanx, stoloniferous vs. rhizomatous. Another intriguing question for forthcoming research is how long it takes to make an association and for how long the association can persist. The role of hormones and other chemical signals in associative learning can be indirectly investigated by restriction of their spread among ramets, for instance by interrupting connections between the ramets.

## Author Contributions

VL designed and performed the experiment. ZM analyzed the data. VL and ZM wrote the manuscript.

## Conflict of Interest Statement

The authors declare that the research was conducted in the absence of any commercial or financial relationships that could be construed as a potential conflict of interest.

## Footnotes

1 http://www.plantanaturalis.com
